# Druggable Targets and Compounds with Both Antinociceptive and Antipruritic Effects

**DOI:** 10.3390/ph15070892

**Published:** 2022-07-19

**Authors:** Hao-Jui Weng, Quoc Thao Trang Pham, Chia-Wei Chang, Tsen-Fang Tsai

**Affiliations:** 1 Department of Dermatology, Taipei Medical University-Shuang Ho Hospital, New Taipei City 23561, Taiwan; wenghaojui@tmu.edu.tw; 2 Department of Dermatology, School of Medicine, College of Medicine, Taipei Medical University, Taipei 11031, Taiwan; chang1994@tmu.edu.tw; 3 International Ph.D. Program for Cell Therapy and Regeneration Medicine, College of Medicine, Taipei Medical University, Taipei 11031, Taiwan; trangpham@ump.edu.vn; 4 Department of Dermatology, Faculty of Medicine, University of Medicine and Pharmacy at Ho Chi Minh City, Ho Chi Minh City 70000, Vietnam; 5 Department of Dermatology, National Taiwan University Hospital, Taipei 100225, Taiwan

**Keywords:** pain, pruritus, neuropathy

## Abstract

Pain and itch are both important manifestations of various disorders, such as herpes zoster, atopic dermatitis, and psoriasis. Growing evidence suggests that both sensations have shared mediators, overlapping neural circuitry, and similarities in sensitization processes. In fact, pain and itch coexist in some disorders. Determining pharmaceutical agents and targets for treating pain and itch concurrently is of scientific and clinical relevance. Here we review the neurobiology of pain and itch and discuss the pharmaceutical targets as well as novel compounds effective for the concurrent treatment of these sensations.

## 1. Introduction

Pain and itch are regarded as two sides of the same coin; painful stimuli normally diminish itch, as it is commonly perceived that “painful” scratching relieves the sensation of itch. Several distinct and specific pathways for pain and itch processing have been discovered, and multiple molecules have been identified in specific neurons involved in the processing of these sensations. Growing evidence indicates that pain and itch have common pathways and properties, including shared mediators and overlapping circuitry in primary afferents. In particular, great overlap in the activity and sensitization of chronic pain and itch is observable. The coexistence of chronic pain and itch is clinically common, such as in psoriasis and atopic dermatitis, two of the most prevalent chronic inflammatory skin diseases, as well as in postherpetic neuralgia [1,2,3]. Some investigations on patients with atopic dermatitis have reported that subjects experiencing concomitant pain and itch accounted for about 59–78% of total cases [4,5]. For plaque psoriasis, one study by Honma et al. showed that 60.3% of patients reported both pain and itch on the skin [1]. A study to measure the impact level of pain and itch on the daily tasks of patients with herpes zoster over 50 years old observed that the proportion of patients having both pain and itch was 67% at inclusion time [6]. Moreover, the percentage of patients suffering from postherpetic neuralgia associated with itch was in the range of 30–58% [3]. Indeed, chronic pain and itch are the most common disorders in clinical practice, and treatments are generally disappointing and unsuccessful [7,8]. Furthermore, persistent pain or itch can generate corresponding disturbances in neural circuitry that pathologically amplify the problem, substantially affecting the quality of life [9]. These matters indicate that common characteristics and pathways in processing itch and pain could be implicated and become a barrier in managing their chronic condition. Therefore, this review will highlight the commonality of two aversive sensations and recent advances in treatments targeting both sensations.

## 2. Overlapping Nature of Pain and Itch

Pain and itch share many overlapping properties in various aspects. In terms of evolutionary significance, both pain and itch sensations are hypothesized to be projective warnings against potential harm by inducing appropriate reflexes and generating aversive responses. Anatomically, both pain and itch are transmitted via the C fibers of primary sensory afferents. Clinically, patients with chronic pain and itch often present with multiple similar manifestations. For example, some patients report attentional focus on the body-related stimuli, which leads to hypervigilance and hypersensitivity via peripheral and central sensitization [10,11,12,13,14,15]. In sum, chronic pain and itch have various similarities. Pruritogens may activate nociceptive primary afferent fibers and generate simultaneous pruritic and nociceptive sensations, as well as body sensations leading to hypersensitivity. They can also induce central and peripheral sensitization [16]. Furthermore, surgical removal of the ventral lateral funiculus of the spinal cord has been demonstrated to attenuate itch and pain concomitantly, and some reports have observed that itch cannot be elicited in patients with congenital insensitivity to pain [17,18,19]. In the past, some cancer patients suffering from intractable pain underwent anterior cordotomy to interrupt the transduction pathway of pain. This classical method resulted in some significant improvement and enhancement of pain thresholds, especially for sharp pain [20,21]. After that, Hyndman et al. observed that cordotomy patients also reported reduced sensation to pruritogens [22]. Interestingly, the attenuated response to itch only occurred in the analgesic regions, and this sensation was produced normally in regions with normal pain [22]. To sufficiently capture the complexity of the pathogenesis of pain and itch, understanding their mechanisms and commonalities is essential. Thus, novel strategies can be developed to target the refractory chronicity of pain and itch.

### 2.1. Overlapping of Algogens and Pruritogens

Multiple peripheral and spinal neurons are involved in the pathogenesis of both pain and itch [18,23,24,25,26,27,28,29]. In fact, some pruritogens can evoke sensations and behaviors associated with both pain and itch [30]. For instance, histamine and capsaicin are at times considered “partial pruritogens”, as these agents can induce a combination of behaviors including both scratching and wiping, depending on how and where they are applied [31,32]. When histamine is applied on the skin, the sensation of itch is perceived. However, when histamine is injected into the underlying tissue, pain is perceived instead. Notably, a psychophysical study by Sikand et al. demonstrated the coexistence of pain and itch when canonical “pruritogens” or “algogens” were administered into human skin [16]. In this study, the intradermal injection of histamine, a canonical pruritogen, induced itch along with transient pain sensations such as pricking and burning. Conversely, the intradermal injection of capsaicin, a canonical algogen, evoked itch along with a pain sensation in a concentration-dependent manner [16]. Other pruritogens may also excite or sensitize a subset of nociceptors, including SLIGRL (a peptide derived from the N-terminus of protease-activated receptor-2), serotonin, acetylcholine, endothelin-1, chloroquine, BAM8-22, prostaglandin-E2, and bradykinin [33]. This evidence demonstrates the overlapping nature of itch and pain and suggests therapeutic targets for the treatment of pain and itch at the same time.

### 2.2. Central and Peripheral Sensitization in Pathological Itch and Pain Conditions

**Figure 1:**
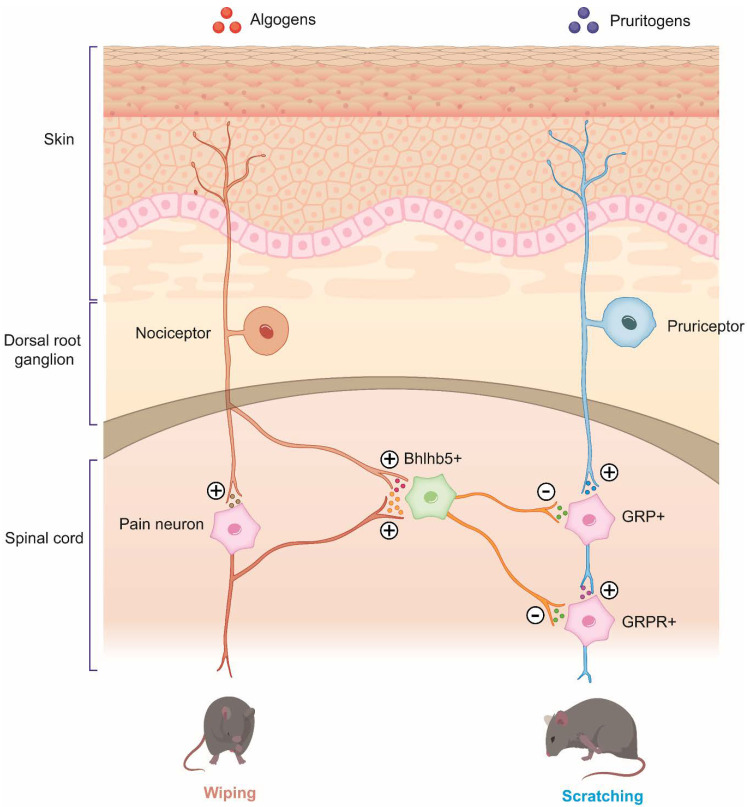
Interaction between pain and itch transmission in physiological conditions. The activation of itch primary afferents in dorsal root ganglia by pruritogens stimulates the release of excitatory neurotransmitters from the terminals of the secondary itch neurons in the spinal cord, leading to the release of GRP and opioids to activate GRPR+ interneurons for itch transmission. Stimulation of nociceptors induces the activation of secondary nociceptive neurons in the spinal cord for nociceptive transduction. Simultaneously, the activation of nociceptors results in the subsequent activation of Bhlhb5+ inhibitory neurons to compress itch transmission in GRPR+ neurons. Furthermore, spinal cord opioids can activate k-opioid receptors to suppress both pain and itch via reducing µ-opioid receptor activity and enhancing the activity of Bhlhb5+ inhibitory neurons. GRPR—gastrin-related peptide receptor; GRP—gastrin-related peptide; Bhlhb5—Class B basic helix-loop-helix protein 5.

**Figure 2:**
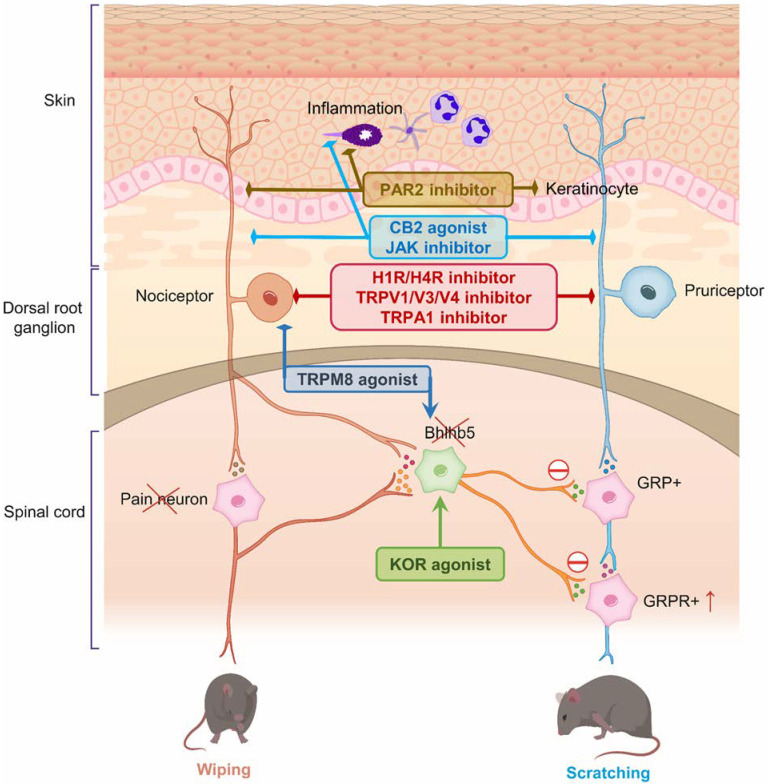
Interaction between pain and itch signaling in pathological (chronic) conditions relating to druggable targets with both antinociceptive and antipruritic effects. Multiple elements can contribute to the development of the mismatch and sensitization of chronic pain and itch in pathological conditions. (1) Afferents involved in pain and itch can be activated by either pruritogens or algogens, leading to the mismatched activation of pruriceptors or nociceptors, and partly account for peripheral sensitization. Moreover, central sensitization of spinal cord neurons is associated with (2) the upregulation of GRP and GRPR, and (3) reduction or loss of inhibitory control from Bhlhb5+ inhibitory neurons. Subsequently, these events disrupt the normal interaction between itch and pain. Arrow—activation; diamond—inhibition.

Under normal physiological conditions, the sensations of pain and itch are considerably distinct (Figure 1). However, under pathological conditions, the chronicity of these sensations blurs the differentiation of these sensations, complicating their treatments (Figure 2). In fact, painful stimuli may be no longer sufficiently strong to inhibit itch and may even potentiate itch in conditions characterized by chronic pruritus because of neural plasticity. Moreover, magnetic resonance imaging study demonstrated that perception of pain and itch is governed by overlapping areas of the brain [34].

Chronic pain and chronic itch involve a similar disruption in the peripheral sensitization of the primary afferent neurons and central sensitization of the central nervous system [35]. This leads to hypersensitivity or the inappropriate hyperactivation of nociceptive and pruriceptive neurons [36,37,38,39,40]. Persistent pain and itch, which are both aversive sensations, have several correlated clinical manifestations. If uncontrolled, they exert a strong negative impact on the patient’s quality of life. Notably, several mediators are engaged in both chronic itch and chronic pain, including opioids, proteases, substance P, nerve growth factor (NGF), neurotrophin 4, and their respective receptors, including µ-opioid receptor (MOR), k-opioid receptors (KOR), protease-activated receptor 2 (PAR-2), tyrosine kinase receptor (TrkA), transient receptor potential (TRP) channels, and cannabinoid receptors [34]. Understanding the similarities between central and peripheral sensitization in chronic itch and chronic pain may aid in the development of effective treatments.

#### 2.2.1. Peripheral Sensitization

The sensitization of the peripheral nervous system may also contribute to chronic itch [41]. The abnormal activation of primary sensory afferents can be partly explained by certain aberrant characteristics, including the hyperinnervation of intraepidermal skin fibers and lower tolerance thresholds in chronic itch and chronic pain [35,42,43,44,45,46]. In the context of chronic itch, common pain-evoking triggers, such as electrical, mechanical, chemical, and heat activators, provoke itch instead of pain in the affected skin regions. Similarly, the classic inflammatory algogens, including bradykinin, serotonin, tumor necrosis factor-alpha (TNF-α), and prostaglandins, may also activate or sensitize pruriceptors and provoke itch [47,48,49].

Notably, elevated nerve growth factor (NGF) levels in the skin and blood have been reported in patients with atopic dermatitis, contact dermatitis, and chronic prurigo, as well as in the pruritic lesions of the patients with psoriasis [50,51,52,53]. Elevated NGF levels have also been observed in painful disorders, including chronic localized pain and vulvar dysesthesia. This finding is consistent with observations that NGF may stimulate nociceptors [34,54]. In addition, NGF may activate peripheral histamine-sensitive C-fibers. These fibers are engaged in the signaling of itch, and the serum levels of NGF are correlated with the clinical severity of atopic dermatitis [50]. Taken together, theoretically anti-NGF therapy might become the treatment target for refractory chronic pain and chronic itch despite its failures in clinical trials on osteoarthritic pain [55,56]. The similarities between localized pain and pruritus reveal the analogous pathogenesis of the activity and the sensitization of nerve endings in peripheral disorders characterized by these two sensations.

#### 2.2.2. Central Sensitization

Chronic pain and chronic itch have similar central sensitization mechanisms. These conditions can result from poor inhibitory control and increased excitatory synaptic transmission. Changes in central sensitization may lead to abnormal sensations, such as allokinesis and hyperkinesis in chronic itch, and allodynia and hyperalgesia in chronic pain. These sensory abnormalities involve the body being sensitized to innocuous mechanical stimuli that do not normally provoke pain and itch [44,57,58,59].

There are parallel phenomena for chronic pain and chronic itch via neuroplasticity changes in the spinal cord. Specifically, allokinesis in chronic itch can occur due to light-touch-elicited itch via Aβ-LTMRs (low-threshold mechanoreceptors) in the adjacent unaffected skin. The maintenance of this activation requires the continual activity of primary C-fibers and nociceptors [60,61]. This phenomenon corresponds to the allodynia that affects the uninjured surroundings of nerve injuries in chronic pain. Another manifestation of hypersensitivity in persistent itch is hyperkinesis, in which a punctate mechanical stimulus, such as a pinprick, triggers an intense itch sensation in regions in the vicinity of the affected itchy skin. Hyperknesis is similar to hyperalgesia, a chronic pain phenomenon in which slightly painful stimulation of the sites adjacent to a lesion induces extreme pain [57,62,63]. These phenomena of hypersensitivity can be partly explained by neural plasticity within the dorsal spinal cord leading to central sensitization via C-fibers and Aβ fibers [64,65,66,67].

Pruritogens are recognized as painful stimuli in some chronic neuropathic pain disorders. Supporting these observations, the application of histamine iontophoresis was perceived as burning pain instead of itch in patients suffering from neuropathic pain. It suggested an abnormal spinal hypersensitivity to C-fiber input in chronic pain [68,69]. In addition, in the context of the neuropathic itch of brachioradial pruritus, some patients reported symptoms resembling neuropathic pain, including burning, stinging, and tingling [70]. Conversely, patients with chronic itch demonstrated an itch reflex to canonical painful stimuli, such as repetitive electrical shock, bradykinin, and acetylcholine [24,44,71]. This mechanism leads to a vicious itch-scratch cycle in patients with atopic dermatitis [72]. Furthermore, painful electrical stimuli, acetylcholine, and bradykinin may provoke itch instead of pain in patients with atopic dermatitis [71,73]. Interestingly, a significant proportion of patients complain about postherpetic itch instead of pain, and more than 25% of patients suffering from postherpetic pain also report itch [3,74,75]. In this respect, medicines for neuropathic pain, such as gabapentin, were also reported to attenuate neuropathic itch [76].

## 3. Therapeutic Targets for Pain and Itch

Considering the overlapping mechanisms of pain and itch, multiple therapeutic targets and their derived compounds exist with both antipruritic and analgesic effects. The existence of such compounds reflects the complex nature of the association in the pathogenesis of pain and itch. Here we enlist the druggable targets for therapeutics effective in both pain and itch, with reviews for novel and relevant compounds in the preclinical testing of these therapeutics (Table 1).

### 3.1. TRPV1 (Transient Receptor Potential Vanilloid 1)

TRPV1 was initially identified as a marker for pain-sensing neurons and remains one of the most investigated channels in the thermoTRP family [77]. It is known to be activated by capsaicin and noxious heat with subsequent neuroinflammatory processes [78]. Mice deficient in TRPV1 demonstrate a drastic reduction in various pain-associated behaviors [79]. Moreover, evidence from rodents and monkeys indicates that TRPV1 is also responsible for histamine-dependent itch [80,81].

Several TRPV1 antagonists have been shown to alleviate both pain and itch. Due to severe side effects from the systemic administration of TRPV1 antagonists, currently, the strategy of TRPV1 channel blockade has been focused on topical applications. One example is asivatrep (PAC-14028), a TRPV1 antagonist, reduces both pain and itch behaviors in animal models. It has been demonstrated that asivatrep reduces pain in inflammatory bowel disease, diabetic neuropathy, and visceral pain [82]. In a mouse model of atopic dermatitis, asivatrep alleviated itch along with decreased mast cell degranulation and neuroinflammation [83]. In a Phase III trial, the topical application of asivatrep reduced itch and skin inflammation in adult patients with atopic dermatitis [84]. Similarly, another TRPV1 antagonist with modified “soft drug” properties, AG1529, demonstrated efficacy in ameliorating histamine-induced pain and itch in animal models [85].

Interestingly, topical capsaicin may also alleviate pain and itch as a TRPV1 agonist, with a proposed mechanism involving the extended desensitization of sensory neurons. For example, NGX-4010, the active ingredient of 8% trans-capsaicin patches, is effective in treating pain in neuropathies [86,87]. Previous studies have also shown that 0.025% and 0.075% capsaicin creams may be effective in the treatment of arthritic pain [87]. Evidence suggests that topical capsaicin is also effective in the treatment of itch in various dermatoses, such as postherpetic pruritus, uremia, notalgia paresthetica, and chronic prurigo [88,89].

### 3.2. TRPV3 (Transient Receptor Potential Vanilloid 3)

TRPV3 is a temperature-sensitive channel expressed in the neuronal and non-neuronal structures of the skin, including sensory neurons, keratinocytes, hair shafts, and vasculature [90]. Although the exact roles of TRPV3 in pain and itch remain to be elucidated, several lines of evidence indicate that TRPV3 is involved at least in the modulation of pain and itch, as well as in the development of dermatitis. Mice deficient in TRPV3 displayed reduced pain response to noxious heat [91]. Transgenic mice carrying a point mutation, Gly573Ser, leading to overexpression in TRPV3 showed increased itch behaviors and peripheral neurite outgrowth in the skin [92]. Therefore, TRPV3 may be an ideal pharmacological target for the concurrent inhibition of pain and itch. However, drug development targeting TRPV3 is sluggish due to a lack of successful compounds progressing into clinical trials. This paper reviews several novel compounds with preclinical data suggesting promise in the concurrent targeting of pain and itch.

One example is citrusinine-II, an acridone alkaloid derived from the plant *Atalantia monophyla* [93]. It is a TRPV3 inhibitor that targets its S4 helix. In the mouse model, citrusinine-II demonstrates antipruritic activities against itch induced by histamine and dry skin, but not acute itch induced by chloroquine. Moreover, citrusinine-II possesses antinociceptive properties against pain evoked by noxious heat and acetic acid [93].

Dyclonine, an over-the-counter oral anesthetic for pain from sore throats, exerts its antinociceptive and antipruritic effects through TRPV3 inhibition [94,95]. Electrophysiological studies have implicated dyclonine as a potent TRPV3 inhibitor under the thermal or chemical activation of TRPV3. In mouse models, dyclonine administration reduced TRPV3-associated pain and itch behaviors. Hydra Biosciences’ FTP-THQ, a selective TRPV3 antagonist, also showed favorable efficacy in relieving histamine-induced itch and formalin-induced pain in rodents [96,97].

### 3.3. TRPV4 (Transient Receptor Potential Vanilloid 4)

TRPV4 is widely expressed in the body, including the sensory neurons, immune cells, skin, vasculature, etc. Notably, it serves as a sensor for osmolarity, as well as chemical, thermal, and mechanical triggers [98]. TRPV4 is known to be involved in pain and itch. Mice deficient in TRPV4 exhibited attenuated pain responses to hypotonicity and formalin [99,100]. They also exhibited attenuated itch responses to chloroquine, histamine, and serotonin [101,102].

TRPV4 blockade has attracted interest for its potential in treating both pain and itch. Isopropyl cyclohexane, along with its derivatives, is a novel compound. They were patented by the National Institute of Natural Science in Japan for their efficacy in preventing pain and itch in hypersensitive skin [103]. A series of compounds have also been patented by the Liedtke’s group to manage pain and itch [103].

### 3.4. TRPA1 (Transient Receptor Potential Cation Channel Subfamily A Member 1)

Since its discovery in 2003, TRPA1 has garnered substantial attention for its potential in pharmaceutical development [104]. Expressed as a subset of TRPV1-expressing sensory neurons, TRPA1 is responsible for chemically, thermally, and mechanically evoked pain and itch. It is also expressed in the non-neuronal tissues such as fibroblasts, pancreas, lung, urinary tract, and endothelium. In several animal studies, TRPA1-deficient mice exhibited attenuated pain responses to formalin, noxious cold, and tactile force [105,106,107,108]. Corresponding evidence was reported in a study of gain-of-function N855S mutation on TRPA1 in patients with familial episodic pain syndrome [109]. In a mouse model, TRPA1 was found responsible for histamine-independent itch by Mas-related G-protein coupled receptor member A3 (MrgprA3) as a downstream effector [108]. A psychophysical study in human observed that a moderate itch sensation was evoked by the topical application of trans-cinnamaldehyde, a TRPA1 agonist [110].

Unlike TRPV1 antagonists, the systemic administration of TRPA1 antagonists does not induce major adverse effects. However, currently, all known clinical trials on TRPA1 antagonists have been discontinued, possibly due to pharmacokinetic issues and poor mouse-to-human translational results from intrinsic species-dependent properties in TRPA1 per se [111]. One potential novel approach for treating pain and itch via TRPA1-mediated mechanisms involves the manipulation of the TRPA1-V1 complex. Tmem100, a regulator of the TRPA1-V1 complex, is associated with both TRPA1-related pain behaviors and itch and was patented for their management [112,113].

### 3.5. TRPM8 (Transient Receptor Potential Cation Channel Subfamily M Member 8)

TRPM8 is a cold-sensing channel in a subset of sensory neurons [114,115]. Several natural and synthetic cooling agents have been identified as its agonists, such as menthol, icilin, rotundifolone, eucalyptol, borneol, etc. [116]. These canonical TRPM8 agonists have been shown to alleviate both pain and itch. Mounting evidence from both human and animal studies indicates that the application of natural TRPM8 agonists such as menthol, eucalyptol, and icilin may attenuate the chronic pain of widely different etiologies. The analgesic effect of TRPM8 agonists is likely due to the desensitization of nociceptive neurons following initial activation. These include inflammatory pain, migraine, and neuropathic pain from chemotherapy, nerve injury, and herpes zoster [117,118,119,120,121,122]. Meanwhile, menthol and peppermint oil have traditionally been employed as antipruritics. A study by Palkar et al. unveiled the neural circuitry for TRPM8-mediated antipruritic effects and provided a solid basis for its mechanism [123].

Aside from canonical TRPM8 agonists, several novel compounds with TRPM8 agonist properties demonstrate great potential for treating both pain and itch. For example, di-isopropyl-phosphinoyl-alkane (DAPA), a synthetic TRPM8 agonist, has been patented for treating sensory dysfunction, including pain and itch [124]. Another synthetic TRPM8 agonist, WS-12, exhibited analgesic effects in animal models and also possesses the potential for treating pruritus [125].

### 3.6. TRPC3 (Transient Receptor Potential Channel Subfamily C Member 3)

Emerging evidence has revealed that TRPC3 is a potential target for both pain and itch treatment. It is expressed ubiquitously; in particular, in the somatosensory system, TRPC3 is expressed in a subset of non-peptidergic neurons. Pharmacological and genetic studies in murine models suggest that TRPC3 is involved in pain and nonhistaminergic itch [126]. Hence, TRPC3 may be another candidate for the development of antinociceptives and antipruritics in the future.

### 3.7. Kappa and Miu Opioid Receptors

Opioid receptors consist of a family of G protein-coupled receptors with various physiological functions. They are widely distributed in the central and peripheral systems, as well as the digestive tract. There are four types of opioid receptors, including μ-opioid receptor (MOR), k-opioid receptor (KOR), δ-opioid receptor (DOR), and opioid receptor-like 1 receptor. Among them, KOR and MOR are known to be involved in sensations of pain and itch. Intrathecal administration of morphine induces itch in 10–20% of obstetric patients [127]. One classic pharmacology study with various opioid receptor blockers by Ko et al. demonstrated that opioid-induced itch is mediated by MOR in the central nervous system [128]. In the skin and associated peripheral nervous system, an imbalance of KOR and MOR has been postulated to signal itch, as the agonists of MOR provoke itch, and the agonists of KOR inhibit itch [129].

The analgesic effects of opioids, including morphine, oxycodone, oxymorphone, and fentanyl, are mediated by MOR. However, opioids also bring about major adverse effects, including addiction and respiratory depression. Given that KOR is not associated with such adverse effects, much effort has been made into the development of selective and peripherally acting KOR agonists for the treatment of pain and itch. For example, difelikefalin (CR845), a peptide-derived, peripherally acting KOR agonist, demonstrates great efficacy for uremic pruritus and postsurgical pain [130,131]. Another novel KOR agonist, HSK21542, has shown promising results for antinociceptive and antipruritic effects in animal models [132]. Similarly, triazole 1.1, a synthetic KOR agonist, displayed both antinociceptive and antipruritic effects in rodents [133].

Several canonical opioids also demonstrate efficacy in the treatment of both pain and itch. Naloxone and naltrexone, the opioid receptor antagonists with the greatest affinity against MOR, have been reported to be effective in the treatment of itch in various diseases [134,135]. The administration of these agents alone has been found to enhance pain [136]. However, when these agents are administered with buprenorphine, a partial MOR agonist, they have treatment effects on pain, particularly in patients with opioid addiction [137]. Another example is butorphanol, a partial agonist and antagonist for MOR and an agonist for KOR. Butorphanol is widely used as an analgesic. Studies have noted that butorphanol may be effective for the treatment of itch [138,139].

Nalbuphine is another promising candidate for the treatment of pain and itch at the same time. As a KOR agonist and partial MOR agonist/antagonist, nalbuphine possesses both antinociceptive and antipruritic properties. It is commercially available for managing pain. Additionally, it has been shown to be effective in uremic pruritus, opioid-induced pruritus, and chronic prurigo in various studies [140,141,142].

### 3.8. Histamine Receptors

Histamine receptors belong to the G protein-coupled receptor family and consist of four types of receptors, H1R, H2R, H3R, and H4R. These receptors are widely expressed in the nervous system, immune cells, fibroblasts, endothelium, epithelium, etc. [143]. The most important physiological functions for histamine receptors are the modulation of inflammation, as well as the signaling of itch circuitry. Histamine has long been known to induce the triple response of Lewis, which is characterized by wheals and inflammatory changes, and intradermal injection of histamine evokes the sensation of itch [144,145]. The prescription of antihistamines has become a standard and traditional treatment for itch, although the effects are at times suboptimal. Among the four receptors, H1R and H4R are most involved in both pain and itch.

#### 3.8.1. Histamine H1 Receptor (H1R)

H1R is an excitatory receptor expressed in sensory neurons and responsible for histamine-dependent itch via mechanical insensitive fibers (C_MIA_) with TRPV1 as a downstream effector [80]. Although not clinically or widely recognized as a common target for analgesics, H1R has long been implicated in the pathogenesis of neuropathic pain in various animal models. Antihistamines targeting H1R, including chlorpheniramine, fexofenadine, and promethazine, have shown efficacy in attenuating pain in different neuropathic pain models in rodents [146]. Several H1R antagonists, such as diphenhydramine, orphenadrine, mepyramine, and pyrilamine, have been reported to be clinically beneficial in the management of severe and, sometimes, intractable pain [147].

#### 3.8.2. Histamine H4 Receptor (H4R)

H4R is expressed in a subset of small-to-medium-diameter sensory neurons and the spinal cord. Pharmacological blockade and the genetic deletion of H4R suggest that the activation of H4R triggers downstream itch signaling [148,149]. Evidence suggests that the analgesic effects of H4R manipulation are dependent on the administration routes. When various H4R antagonists were administered intrathecally in neuropathic pain models, pronociceptive effects were observed. These results are supported by the observation that the intrathecal administration of H4R agonists (ST-1006 and VUF8430) attenuates neuropathic pain [146]. By contrast, the intraperitoneal or subcutaneous administration of H4R antagonists, including TR-7 and JNJ7777120, alleviates pain in nerve injuries or inflammation in murine models [150,151]. Although JNJ7777120 displayed both analgesic and antipruritic effects in an experimental setting, its short half-life prevented its entry into clinical development. Nevertheless, this peripheral blockade approach for H4R seems promising for treating pain and itch at the same time.

### 3.9. Cannabinoid Receptors

Cannabinoid receptors belong to the G protein-coupled receptor family and are involved in multiple physiological processes, such as inflammation, cognition, itch, and pain. There are two identified receptors, cannabinoid receptor 1 (CB1) and cannabinoid receptor 2 (CB2), with the former mediating the modulatory effect in both the central and peripheral nervous system, and the latter only in the peripheral nervous system [152,153,154]. CB1 and CB2 share 44% of their sequence homology and are coupled with Gi/o downstream inhibitory signaling pathways [155].

CB1 and CB2 agonists may mediate antinociceptive and antipruritic effects, and a wide range of compounds have been tested for such effects [156]. The primary effects on pain and itch take place mainly by mediating the activities on the sensory neurons. These effects are partly produced by mediating the inflammatory process. Aside from the nervous system, CB1 and CB2 are also expressed in mast cells, macrophages, and the keratinocytes in the skin [157]. Therefore, some of the effects are mediated by the modification of immune cells and barriers in the skin.

The agonists of CB1 have been employed in the treatment of both pain and itch. However, due to disturbances in physiological functions caused by its activities on the central nervous system, CB1 agonists may bring about major side effects, such as hallucination and panic [158]. Given that CB2 is mainly distributed in the peripheral tissues, the administration of CB2-selective agonists may theoretically circumvent these side effects through CB1 activation. However, to date, most of the compounds with antinociceptive and antipruritic properties in humans have been found to be nonselective to CB2. One example is dronabinol (delta-9-tetrahydrocannabinol), a synthetic CB1 and CB2 agonist, which has been efficacious in treating intractable itch and chronic pain [159,160].

One practical strategy for avoiding the adverse effects of systemic cannabinoid agonists is via topical administration. However, although the topical administration of various cannabinoid agonists, including HU-210 and anandamide, has been reported for the treatment of itch [161,162,163], whether similar topical approaches exert antinociceptive effects in human remains unclear. Comprehensive investigations are warranted.

### 3.10. Oncostatin M (OSM)

Oncostatin M is a member of the IL-6 family and is a cytokine involved in organ development, inflammation, itch, and pain. Its receptor, oncostatin M receptor (OSMR), is expressed broadly in neural and non-neural cells. OSMR was initially implicated as a receptor for pain. In the sensory neuron, OSMR overlaps with TRPV1 and purinergic receptor P2X3 as a subset of nociceptive neurons [164]. OSMR-knockout mice displayed attenuated pain behaviors [165]. In the murine model, the administration of OSM enhanced hyperalgesia via prolonged ERK signaling and, eventually, led to the priming of chronic pain [166]. Recent investigations on OSMR reveal that it plays more important roles in itch sensitization. The itch intensity of chronic prurigo was found to be correlated to the level of OSM and OSMR-expressing cells in the skin [167]. A study by Tseng and Hoon showed that OSMR is responsible for itch sensitization, and OSM is upregulated in various inflammatory dermatoses and cutaneous T-cell lymphoma [168]. Nevertheless, currently, there is no commercially available agent targeting OSM-OSMR in the management of both pain and itch. In line with the theory that OSM-OSMR is involved in the pathogenesis of both pain and itch, the pharmacological blockade of this axis may lead to the treatment of both sensations in the future.

### 3.11. JAK-STAT (Janus Kinase/Signal Transducer and Activator of Transcription) Pathway

The JAK-STAT pathway was first identified in 1992. Over the past decade, substantial advances in the use of JAK-STAT inhibitors for treating various disorders have been made [169,170]. Currently, four members of the JAK family have been identified, namely JAK1, JAK2, JAK3, and TYK2, with seven members in the STAT family, including STAT1, STAT2, STAT3, STAT4, STAT5a, STAT5b, and STAT6 [171]. JAK-STAT signaling has been implicated widely in different physiological functions, including immunity, cell division, and cancer, as well as in pain and itch. Notably, there is shared JAK-STAT signaling in the neural and immune pathways, contributing to its roles in pain and itch. Key mediators in this process include IL-4, IL-13, Th1, Th2, and Th17. Moreover, both JAK1 and JAK2 have been found to be directly involved in the neural pathway for itch [172,173,174]. JAK-STAT inhibitors, including abrocitinib (JAK1 inhibitor), baricitinib (JAK1 and JAK2 inhibitors), ruxolitinib (JAK1 and JAK2 inhibitors), and upadacitinib (JAK1 inhibitor), can inhibit itch rather rapidly [175]. Moreover, mounting evidence from both basic and clinical studies indicates that JAK-STAT3 signaling plays a role in nociception. JAK-STAT3 signaling is crucial for microglia and astrocyte activation following nerve injuries, which subsequently contributes to pain [176,177]. Another proposed mechanism is that JAK-STAT inhibitors exert their antinociceptive functions by modulating pain-associated mediators such as IL-17, IL-6, and IL-23 [178]. Clinically, baricitinib and upadacitinib have been reported to alleviate pain and associated behaviors in rheumatoid arthritis patients [179].

### 3.12. Nerve Growth Factor (NGF)

NGF belongs to the neurotrophin family and is critical for the growth and maintenance of neurons. There are two receptors for NGF, including a high-affinity tyrosine kinase receptor, tropomyosin kinase receptor A (TrkA), and a nonselective and low-affinity p75 pan-neurotrophin receptor (p75NTR). The binding of NGF to TrkA leads to the activation of Ras/Raf/mitogen-activated protein kinase (MAPK), phosphatidylinositol 3-kinase (PI3K)/Akt, and phospholipase C-γ (PLC-γ) pathways, thus promoting the survival of cells. However, the absence or decreased expression of TrkA can induce the activation of p75NTR, which may trigger the JNK pathway and induce cell apoptosis [180,181]. Studies have indicated that the ratio of TrkA and p75NTR expressed on cell surfaces contributes crucially to the determination of NGF on cells [182,183]. Besides nervous systems, NGF has also been reported to be produced and utilized by other cell types, such as epithelial cells, endothelial cells, mast cells, and B and T cells. In sum, NGF plays a substantial role in the crosstalk among the nervous system, immune system, and other cell types [184,185].

Elevated NGF levels have been implicated in pain and itch for various disorders. In chronic prurigo, atopic dermatitis, and allergic contact dermatitis, NGF overexpression is accompanied by the hyperinnervation of skin nerves, which may cause itch or pain in the lesional skin [51,52,186]. These findings indicate the potential of NGF as a target to treat pain and itch in several diseases. Multiple anti-NGF monoclonal antibodies, including tanezumab and fasinumab, have been developed for the treatment of osteoarthritic pain, low back pain, and pain from other diseases. In theory, this approach may attenuate chronic pain and itch. However, severe adverse effects, such as osteonecrosis, have been reported, which suggests great risk in the development of anti-NGF antibodies, and, thus, many of these developments were halted [187,188].

### 3.13. Protease-Activated Receptor 2 (PAR2)

The protease-activated receptor (PAR) is a G protein-coupled receptor family that requires the proteolytic cleavage of the extracellular N-terminal domain to reveal a new cleaved N-terminus, which can function as a ligand to activate the PARs [189]. There are four members of the PAR family: PAR1, PAR2, PAR3, and PAR4. Among them, PAR2 is widely discussed for its role in both neural and immune systems, as well as its ability in triggering pain and itch [190,191]. PAR2 can be cleaved and activated by trypsin and mast cell tryptase. Research indicates that proteases from specific pathogens, such as house dust mites and some bacteria, can also induce the activation of PAR2 [192]. The expression of PAR2 is broadly seen on various cell types, including neurons, epithelial cells, keratinocytes, mast cells, and neutrophils. This suggests the integral role PAR2 plays in diseases related to these cells.

The role of PAR2 in pain is well established. Conversely, there have been mixed results regarding the role of PAR2 in itch, as current evidence indicates that PAR2 mediates itch via keratinocytes, not directly via sensory neurons [193,194]. Studies have revealed that the activation of PAR2 may contribute to several diseases, such as airway and lung inflammation, arthritis, skin diseases, neurological disorders, and chronic pain [195,196,197]. Thus, PAR2 antagonists were developed to treat these conditions, as well as to treat pain and itch. Animal experiments have supported the premise that PAR2 is a mediator of cancer pain, while the overexpression of PAR2 in atopic dermatitis mouse models induces a severe itch response [198,199,200]. Although various PAR2 antagonists, including peptides, pepducins, small molecules, and antibodies, have been introduced, only some of them have been tested for treating pain and itch. For example, FSLLRY-NH2 was proven to inhibit neuropathic pain in rats with spinal cord injury, and reduce dermatophyte-associated in atopic dermatitis mouse models; a PAR2 antibody, MEDI0618, is undergoing a Phase I clinical trial, with an aim for the treatment of chronic pain in the future [194,201,202,203].

### 3.14. Other Agents with Analgesic and Antipruritic Effects

Multiple agents with both analgesic and antipruritic effects are available now and are commonly prescribed clinically. Although the targets and mechanisms for many of them are not fully understood, they are discussed as follows because they are of undeniable scientific and clinical values.

#### 3.14.1. Botulinum Toxin

Botulinum toxin is a neurotoxin produced by the bacterium *Clostridium botulinum*. There are seven types of botulinum toxins, with type A and type B toxins being utilized medically. In the synapses, these toxins disrupt the functions of the SNARE complex and prevent the release of neurotransmitters and, thus, interrupt pain and itch signaling. Botulinum toxin A has been employed in the treatment of various pain conditions, such as migraine, trigeminal neuralgia, postherpetic neuralgia, diabetic neuropathy, occipital neuralgia, complex regional pain syndrome, etc. [204,205]. Botulinum toxin A may also alleviate itch in various disorders, including notalgia paresthetica, keloid, multiple sclerosis, brachioradial pruritus, and Fox–Fordyce disease [206,207,208,209,210,211].

#### 3.14.2. Local Anesthetics

In addition to their known antinociceptive effects, topical anesthetics are most widely used for the treatment of itch. The topical application of lidocaine has been reported to be effective in the treatment of different pruritic disorders, including liver disorders, burns, and other disorders when combined with ketamine and amitriptyline [212,213,214]. Lidocaine is also available as an over-the-counter agent for topical usage. Topical pramoxine has also been found to be effective for the treatment of uremic pruritus [215].

#### 3.14.3. Gapapentin/Pregabalin

Gapapentin and its successor, pregabalin, have been approved for the treatment of painful conditions such as postherpetic neuralgia, spinal cord injury, diabetic neuropathy, and fibromyalgia. The exact mechanisms of gabapentin and pregabalin are still unclear. It is proposed that these two drugs attenuate itch and pain by inhibiting the α2δ unit in voltage-gated calcium channels on sensory neurons and the spinal cord [216]. In the meantime, gabapentin and pregabalin have also been reported to be effective in treating itch for various conditions. These disorders include uremic pruritus, brachioradial pruritus, chronic prurigo, spinal cord injury, and pruritus of unknown origin. [217].

## 4. Conclusions

Pain and itch are distinct sensations yet share some overlapping neural pathways and key molecules in their pathogenesis. Clinically chronic pain and chronic itch may be present at the same time in various disorders. Here we reviewed the common grounds of pain and itch and, at the same time, druggable targets for developing agents to treat pain and itch. Clearly, heterogeneity exists in pain and itch with respect to their molecular signatures, their pathways from the skin to the nervous system, and even in their manifestations for various disorders. The complex nature thus renders their pathogenesis difficult to understand and the creation of pharmacological agents unlikely to fully attenuate the symptoms based on the pathophysiology. With respect to this, it may be reasonable not to focus solely on the symptoms of pain and itch per se but on the symptoms in specific diseases with due regard to the heterogeneity of their pathophysiology. Further investigation is warranted to dissect the differences and similarities in pain and itch pathways, including skin biopsies, functional imaging, and psychophysics, along with techniques such as mouse genetics and in vivo imaging. This will unveil the potential of novel agents with both antinociceptive and antipruritic properties.

## Figures and Tables

**Table 1:** Targets and therapeutic compounds with antinociceptive and antipruritic effects. Abbreviations: KOR—k-opioid receptor; MOR—µ-opioid receptor; H1R—histamine H1 receptor; H4R—histamine H4 receptor; CB1—cannabinoid receptor type 1; CB2—cannabinoid receptor type 2; NGF—nerve growth factor; PAR2—protease-activated receptor 2.

Target	Effects on Itch and Pain	Therapeutic Compounds
TRPV1	↑ pain; ↑ itch	Asivatrep, AG1529, NGX-4010
TRPV3	↑ pain; ↑ itch	Citrusinine-II, dyclonine, FTP-THQ
TRPV4	↑ pain; ↑ itch	isopropyl cyclohexane
TRPA1	↑ pain; ↑ itch	
TRPM8	↑↓ pain; ↑ itch	Di-isopropyl-phosphinoyl-alkanes, WS-12
TRPC3	↑ pain; ↑ itch	
KOR	↓ pain; ↓ itch	Difelikefalin, HSK21542, triazole 1.1, butorphanol, nalbuphine
MOR	↓ pain; ↑ itch	*Buprenorphine–naloxone*, nalbuphine
H1R	↑ pain; ↑ itch	Chlorpheniramine, fexofenadine, promethazine, diphenhydramine, orphenadrine, mepyramine, pyrilamine
H4R	↓ pain(central); ↑ pain(peripheral); ↑ itch	JNJ7777120
CB1	↓ pain; ↓ itch	Dronabinol
CB2	↓ pain; ↓ itch	Dronabinol
Oncostatin M	↑ pain; ↑ itch	
JAK-STAT signaling	↑ pain; ↑ itch	Baricitinib, upadacitinib
NGF	↑ pain; ↑ itch	
PAR2	↑ pain; ↑ itch	FSLLRY-NH2

## Data Availability

Not applicable.
